# Supporting Third Year Medical Students' Skill Acquisition and Self-Efficacy with Coping Models and Process Feedback during Laparoscopic Knot Tying Simulation

**DOI:** 10.3389/fpsyg.2017.01171

**Published:** 2017-07-18

**Authors:** Michael S. Dempsey, Douglas F. Kauffman

**Affiliations:** Boston Medical Center, Boston University Boston, MA, United States

**Keywords:** modeling, feedback, self-efficacy, FLS training, surgical education, laparoscopic knot tying

## Abstract

**Background:** During the third year general surgery clerkship, medical students are required to develop laparoscopic knot-tying skills. Knot-tying skills studies often rely on objective variables (e.g., time, materials used, number of iterations) that lend themselves to correlational analysis of pre- and post-intervention skill level. This study differs by examining how instructional interventions—role modeling and feedback—affect medical students' skill acquisition and self-efficacy during a laparoscopic surgical simulation training session.

**Methods:** Seventy-eight surgical clerkship students were assigned randomly to one cell of a 2X2 factorial design. Participants observed one of two types of role modeling (expert vs. coping) and received either process-oriented or outcome-oriented feedback during a 30-min laparoscopic training session. Participants also completed several surveys that assessed their interest in surgery and their self-efficacy for laparoscopic knot tying.

**Results:** Coping model groups tended to perform better on the knot tying task, though this was less the case in the presence of outcome feedback. Expert model groups slightly outperformed the coping model group on the peg transfer task, but in the presence of outcome feedback they reported the lowest satisfaction with their performance and the lowest self-efficacy for the knot tying task. The coping model combined with process feedback had a positive influence on students' efficiency in learning the task, on their satisfaction with their performance, and on their self-efficacy for laparoscopic knot typing.

**Conclusions:** Results are discussed relative to self-regulated learning theory.

## Introduction

For over three decades, educational researchers have been examining how students' motivation and personal beliefs impact their learning (McCombs, 2017). Research findings over this time period suggest, in short, that ability alone is insufficient for achieving academic success. Some scholars characterize academic success as the product of sustained motivation along with skill development, a distinction more commonly referred to as *will* and *skill* (Weinstein and Mayer, 1986; Weinstein and McCombs, 1998). *Will* refers to a motivational state that moves individuals to engage in learning activities. *Skill* refers to task competencies that develop as the result of training and practice. Skill acquisition can be measured as differences in performance on critical tasks pre- to post-intervention. Individuals' motivation for task-related performance can be gauged with self-efficacy scales in the same manner. Self-efficacy has been shown to predict engagement and persistence in learning activities, traits that are central to motivation. Our goal in the present study was to better understand how learners' task performance and self-efficacy may be affected by modeling and feedback, two sources that inform individuals' self-efficacy.

Perceived self-efficacy refers to an individual's beliefs about his or her ability to perform a task at a specified level of proficiency (Bandura, 1997). When individuals assess their self-efficacy for completing a task, laparoscopic knot typing for example, they are typically asked to make a probability judgment (from 0 to 100) of the likelihood that they can complete that task with a specified degree of proficiency (“What is the probability that you can laparoscopically tie a knot within 2 mm of the target without breaking the suture?”). Self-efficacy is important within surgical education because beliefs about ability mediate and direct a learner's engagement, effort, persistence, and performance on tasks that are critical to success within specific domains. Simply put, individuals with high self-efficacy are generally more engaged, will persist in the face of challenge, use appropriate strategies, and tend to perform at higher levels than peers with lower self-efficacy. Individuals' self-beliefs, then, are critical to understanding and improving their task performance.

Self-efficacy develops through an individual's interactions with and feedback from the environment. Most researchers agree that individuals take information from four sources of information to make their self-efficacy judgments, including prior experiences, role modeling, teacher feedback, and physiological and psychological state (e.g., Zimmerman and Bandura, 1994; Bandura, 1997; Usher and Pajares, 2009; Bruning and Kauffman, 2015). The present study assesses how two of these sources of information—role modeling and feedback—impact third year medical students' laparoscopic knot-tying skill acquisition and self-efficacy during FLS training.

According to Bandura and others (e.g., Schunk, 2003; Artino et al., 2011; Schraw and Gutierrez, 2015). Modeling is critical to advancing learning while promoting self-efficacy (cf. Klug et al., 2011; Lajoie et al., 2015). Role models are effective because they demonstrate constituent skills that lead to successful performance, which raises learners' expectations of their own success, and provides them with motivational incentives. Research has shown, however, that models vary in effectiveness (Schunk and Zimmerman, 2006; Schunk, 2008). Kisantas et al. (2000), for example, proposed that *coping* models will be more helpful to learners than *mastery* models because coping models, “begin by making many errors but gradually are able to identify and eliminate them” (p. 661). We define a coping model as an individual who demonstrates a skill and struggles at first but through persistence and effort begins to demonstrate successful problem solving, task-completion, and increased confidence through positive self-talk. A mastery model, in contrast is defined as an individual who performs a task calmly and Flawlessly from the outset. Zimmerman and Kisantas (2002) found that college students were more successful observing a coping model than when observing a mastery model, or no model at all. In line with this theoretical perspective, we hypothesized that medical students would learn more and report the greater self-efficacy for performing laparoscopic knot-tying tasks when working with a coping model than when working with a mastery model.

Learning solely through observation is not always productive, however (Denton et al., 2008; Shute, 2008). Individuals also need feedback that helps them reflect on how closely their performance matches that of the model. Different feedback types can be used but they vary in the degree to which they influence successful behavior (Hogarth et al., 1991; Shute, 2008; van de Ridder et al., 2015). Process feedback, according to Kulhavy (1977), becomes a “form of new instruction, rather than informing the student solely about correctness” (p. 212). It communicates to individuals the *distance* between their current performance and the performance intended (Hattie and Timperley, 2007). Outcome feedback differs from process feedback by providing *performance-specific* information, which research suggests may have little long-term effect, particularly on novice learners (Lhyle and Kulhavy, 1987). Research with medical students has also supported the beneficial effects of process feedback compared to outcome feedback (Harks et al., 2014). We hypothesized that process feedback would prove more beneficial than outcome feedback to medical students learning Fundamentals of Laparoscopic Surgery.

This study focuses on learner motivation as measured by perceived self-efficacy and how modeling and feedback supports skill development. Third-year medical school interns were randomly assigned into conditions with either a coping or a mastery model and in the presence of either process or outcome feedback.

Our research questions were:

1. How does the presence of a coping model affect medical students' skill acquisition and self-efficacy as compared to the presence of a mastery model?
2. How does the presence of process feedback during a surgical simulation task impact skill acquisition and self-efficacy as compared to the presence of outcome feedback?
3. How do different types of modeling and feedback interact to affect medical students' skill acquisition?

## Methods

### Participants and design

**Figure 1:**
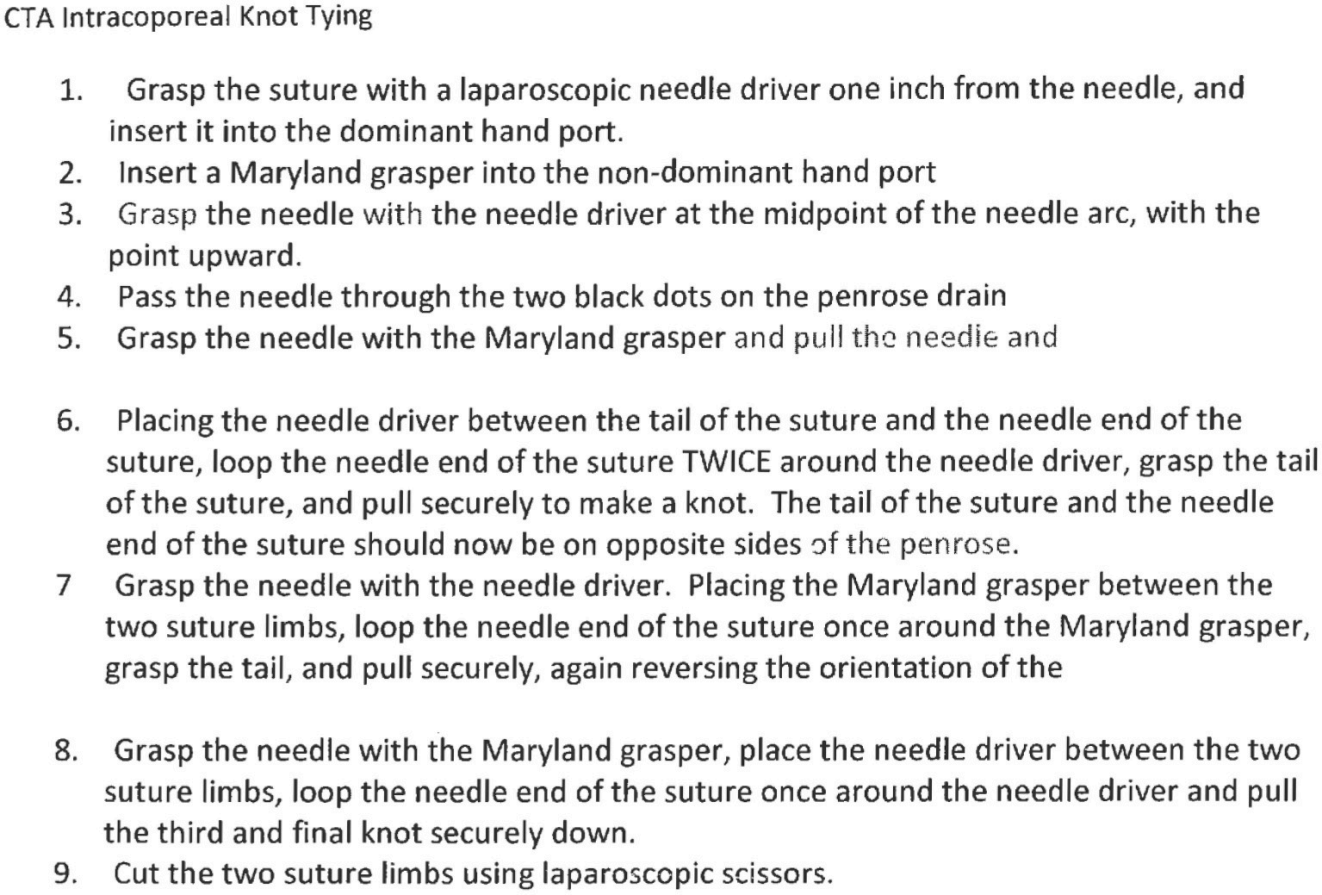
Cognitive task analysis of intracoporeal knot tying.

We employed an explanatory sequential mixed methods research design (Creswell, 2015). In this type of study, the researchers begin by conducting a quantitative study and then supplements the quantitative results with qualitative data. In our case, 78 third year medical students enrolled in a surgical clerkship were assigned randomly to one cell of a 2X2 factorial design (See Figure 1). The first factor was feedback. Students received either process or outcome-based feedback. The second factor was modeling. Students were exposed either to a mastery (expert) or to a coping model. Once the quantitative data was collected, we supplemented our results with targeted interviews of a representative sample from each group.

Our dependent variables included time to complete the knot typing task, the number of knots tied, distance from the target, satisfaction with their performance, and self-efficacy (pre- and post-test). We also asked students to complete a laparoscopic peg-transfer task prior to being trained to tie laparoscopically. In this task, participants used laparoscopic needle drivers to transfer pegs from a board on one side of a laparoscopy simulator to a board on the other side of the simulator. This “pre-test” measure simulates the skills needed to tie knots laparoscopically enabling us to establish that groups had approximately the same skill-set going in to the training.

### Materials

Materials included a pre-experimental survey, that elicited demographic information and self-efficacy for laparoscopic knot tying (e.g., How confident are you that you can grasp the needle with the Maryland grasper. See the **Appendix**), and completed the same self-efficacy survey post-experiment. At post-experiment, participants also completed a satisfaction survey in which they rated their satisfaction with their performance for different learning contexts they had experienced (e.g., An anatomy lab exercise or A suture knot tying activity. See the **Appendix**). Additional materials included: (1) Maryland dissectors used for suturing and fine separation of tissue; (2) pegboards with rubber ring objects used during simulation to practice manipulating instruments; (3) needle drivers, knot pushers, 2-0 silk suture of 90 cm length, endoscopic scissors used to stitch and knot sutures; (4) a penrose drain left in place after surgery; and (5) suture blocks, which hold the penrose drain in place. All materials are consistent with the FLS Manual Skills Written Instructions and Performance Guidelines (2014). Finally, we constructed a brief interview that we used to better understand participant's experiences with the curriculum and instructor. A small subset (*n* = 8) of participants was interviewed by one of the investigators. Interviewees were representative of the entire sample.

### Procedures

Procedures were divided into a series of six phases that included, recruitment, informed consent/survey, peg-transfer task, instruction (experimental phase), practice, post-testing, and interview phases. The recruitment phase consisted of one of the researchers visiting the surgical clerkship during a required lecture to describe the study and invite students to participate. Students were told the purpose of the study was to introduce them to intracoporeal knot tying and to provide an opportunity to practice tying laparoscopic knots in the simulation center. The students were told that laparoscopic knot tying was increasingly important skill for all medical students, particularly those interested in general surgery. A sign-up sheet was passed around the room that listed available timeslots for students to participate in the 60-min training session that occurred outside of class. A maximum of five students were recruited for each available time slot as this was number of available laparoscopic simulators. Upon arrival, participants were asked to read and sign the informed consent and then to complete the pre-experimental survey. Once all participants at each session had completed the survey, they were directed to the FLS simulation room for the peg transfer task.

After the instructor introduced the equipment and modeled how to complete the peg transfer, participants were told, “The object of this task is to transfer the pegs from one side to the other (and then back again?) I will be timing each of you so as soon as you complete the task, please raise your hand. Ready? Begin!!” Participants were given up to 3 min to transfer pegs from one side to the other and back again. A research team member timed how long it took each participant to complete the peg transfer and recorded it on the pre-experimental survey. Once the allotted 3 min was over all participants still working were given a maximum score of 180 s and asked to turn their attention to the instructor.

Next, the instructor provided a brief lecture and demonstration on how to tie laparoscopically. Figure 1 provides a Cognitive Task Analysis of this activity. For groups in the Mastery model condition, the instructor demonstrated the steps flawlessly and with little effort. Groups exposed to the coping model, in contrast observed the same instructor intentionally make several mistakes common to those not previously exposed to laparoscopy. Specifically, the instructor dropped the needle driver and struggled with grasping it but talked herself through the steps and eventually demonstrated mastery.

After the lecture, participants were given up to 10 min to complete the intracoporeal knot tying activity and were exposed to either process or outcome feedback. The process feedback group received feedback on the things they were doing well and things they needed to work on. The emphasis of this feedback was hard work, effort, and improvement over time. The outcome feedback group, in contrast were provided feedback only about the outcome (e.g., “you have successfully tied 2 knots.”) After completing the post-experimental survey, students were debriefed, invited to participate in an interview at a later date, and dismissed.

Finally, two participants from each experimental group (*n* = 8) were recruited to participate in a brief four question interview following the post-experimental survey. Respondents were asked (a) their overall impressions of their training, (b) how they experienced the instructor's knot tying example, (c) how they viewed the feedback they received, and (d) what (if anything) they planned to do moving forward (e.g., more training).

## Results

Means and standard deviations for each group on the pre- and post-experimental self-efficacy, satisfaction, peg-transfer time, and knot tying time are presented in Table 1. Pre- and post-test means and gain scores for self-efficacy are presented in Table 2. In both modeling conditions, participants showed greater self-efficacy at post-test for both feedback conditions no matter the modeling they received. We conducted a multivariate analysis of variance with pre-experimental self-efficacy for knot tying and peg transfer time as dependent variables. The purpose of this MANOVA was to learn if groups possessed equivalent self-efficacy and skill levels prior to participating in the experiment. There were no significant differences between groups at pre-test on peg transfer time or knot tying. Results of this MANOVA revealed an unexpected main effect for feedback *F*_(2, 73)_ = 11.84, *p* < 0.01. At the multivariate level, Box's test of equality of covariance matrices was non-significant, *F* = 2.96, *p* = 0.123, indicating that the assumption of homogeneity of covariance matrices was not violated. We further investigated the main effect finding with univariate analyses. Results revealed a main effect for feedback on the pre-experimental self-efficacy scale *F*_(1, 74)_ = 19.46; *Mse* = 206.88; partial eta sq = 0.148. Participants in the outcome feedback group had significantly higher self-efficacy (*M* = 64.34; *SD* = 12.47) than did participants in the process feedback group (*M* = 50.15; *SD* = 16.31). Though unexpected, we attribute this finding to randomness. It is possible that our relatively small sample size contributed to the observed difference between groups. We predicted that process feedback would lead to higher self-efficacy at post-testing, reducing concerns that pre-test differences would influence post-test results.

**Table 1:** Means and standard deviations for dependent variables at post-test.

**Modeling**	**Feedback**	**Pre-SE**		**Peg time**		**Knot time**		**Satisfied**		**Post-SE**	
		***M***	***SD***	***M***	***SD***	***M***	***SD***	***M***	***SD***	***M***	***SD***
Expert	Outcome	60.9	14.47	152.2	28.7	593.9	16.3	56.0	12.7	56.6	12.7
	Process	53.0	15.14	146.5	25.6	579.1	28.6	59.4	16.9	81.5	10.5
Coping	Outcome	68.2	11.34	167.6	24.5	562.2	40.8	83.4	16.0	81.0	13.6
	Process	47.3	17.3	155.3	34.3	441.1	35.3	81.0	19.7	80.2	9.0

**Table 2:** Means and standard deviations for dependent variables at post-test.

**Modeling**	**Feedback**	**Pre-SE**	**Post-SE**	**Gain**
		***M***	***M***	***M***
Expert	Outcome	60.9	56.6	4.3
	Process	53.0	81.5	28.5
Coping	Outcome	68.2	81.0	12.8
	Process	47.3	80.2	32.9

We conducted a MANOVA with knot tying time, satisfaction, and post-experimental self-efficacy as the dependent variables. Results of this analysis revealed main effects for modeling *F*_(3, 72)_ = 21.45, *p* < 0.01; partial eta *sq* = 0.038 and for feedback *F*_(3, 72)_ = 12.68, *p* < 0.01; partial eta *sq* = 0.875. Additionally, our results revealed a significant modeling by feedback interaction. *F*_(3, 72)_ = 18.36, *p* < 0.01; partial eta *sq* = 0.948. We followed this analysis up with a series of univariate analyses for knot tying time, satisfaction and post-experimental self-efficacy respectively.

For knot tying time, our univariate analysis revealed main effects for modeling *F*_(1, 74)_ = 42.47, *p* < 0.01, a main effect for feedback *F*_(1, 74)_ = 27.28, *p* < 0.01 and a significant modeling by feedback interaction *F*_(1, 74)_ = 16.70, *p* < 0.01. Participants who received process feedback took significantly less time to complete the knot tying task than those who received outcome feedback, but only when they observed coping models.

On post-experimental self-efficacy scale, we observed a main effect for modeling *F*_(1, 74)_ = 20.25, *p* < 0.01, a main effect for feedback *F*_(1, 74)_ = 21.86, *p* < 0.01 and a significant modeling X feedback interaction *F*_(1, 74)_ = 24.66, *p* < 0.01. As seen in Table 1, those participants who observed an expert model and received outcome feedback had significantly lower post experimental self-efficacy than participants in the other three groups.

For participant satisfaction (“How satisfied are you with your performance?”), we found a significant main effect for modeling *F*_(1, 74)_ = 21.41, *p* < 0.01. Participants who received coping models reported that they were significantly more satisfied with their performance than were those who received expert models.

Finally, a member of the research team interviewed a subset of eight participants—two from each experimental group—about their experiences with the FLS training they had just completed. Three open ended questions were asked. First, we asked participants their overall impressions of the training. All participants were generally positive and expressed enthusiasm for the opportunity to complete the training. For example, one participant stated, “This was a great experience. It was helpful to see it done by an expert first. I really liked the peg transfer task too.” Another participant stated, “It was very valuable.” Second, we asked participants specifically about the instructor's modeling of the procedure. Comments from the mastery model group included, “it was good” and “it made me see how to do it correctly,” whereas comments from participants who observed the coping model included, “At first when I was practicing, I dropped the needle and got frustrated. Then I remembered that (the instructor) dropped the needle as well and was able to pick it up and continue. That helped me” and “Watching (the instructor) talk through the steps helped me to focus on what I had to do when I was practicing. It was very helpful” Third, we asked participants to discuss how the feedback they received impacted them. Again, whereas those who received outcome feedback reported generally positive feelings, participants who received process feedback reported that they were better able to regulate their own learning and felt more confident that they could transfer what they learned to an authentic surgical environment. Generally, participants who observed coping models and received process feedback reported seeing the model plan, act strategic, and monitor herself and that this had an impact on their immediate and future behavior. One student, for example, stated, “getting feedback on the things I was doing well and the things I needed to work on was really helpful. Knowing I was doing some things right helped me stay confident even when I dropped the needle. It helped me think about and plan what I needed to do in order to improve.”

## Discussion

The purpose of this study was to better understand how third year medical students' skill development and self-efficacy might be affected by different types of modeling and feedback. We used peg transfer time, knot tying time, satisfaction with performance, and post-self-efficacy as dependent variables of skill acquisition. Self-efficacy, a key component of learner motivation, was measured pre- and post-experiment. Specifically, we asked three questions: (1) Are skill acquisition and self-efficacy differentially affected when exposed to a coping vs. an expert model? (2) Are skill acquisition and self-efficacy differentially affected if receiving process feedback vs. outcome feedback. (3) Finally, do modeling and feedback interact to affect medical students' skill acquisition?

Our results show that different model and feedback strategies affect medical students' learning laparoscopic knot tying differently and that there is an interaction between model type and feedback type. Related to model type, peg time was shortest for students exposed to an expert model and given outcome feedback, but they also reported the lowest satisfaction with their effort and the lowest self-efficacy. Furthermore, knot time for the expert groups was notably longer in both feedback conditions than it was for the coping groups. The coping groups, by contrast, reported higher satisfaction and self-efficacy pre- to post-experiment with both feedback types, and they were able to complete the knot tying task more quickly than the expert group in both feedback conditions. It is possible that the coping groups took more time to complete the peg transfer because the coping model emphasized the role of mistakes and frustration as part of the learning process. Students may have taken longer on this initial task—challenging because of its novelty and difficulty—because they focused on doing learning the task rather than on doing the task correctly in as little time as possible. Our qualitative results suggest that this is indeed how students felt and behaved. Our results also show that different feedback strategies affect medical students' learning differentially. In the expert groups, both peg time and knot time were lowest for the process group than for the outcome group. This was the case for the coping groups as well.

### Significance of the study

These findings are important for both theoretical and practical reasons. From a theoretical perspective, results from this study are consistent with previous research on both models and feedback. Zimmerman and Kisantas (2002), for example, found that the effectiveness of feedback and modeling are dependent on learners' prior knowledge, with novice learners responding more positively to coping models and process feedback. Our findings replicate this in a new context and with different population of learners. Will and skill are necessary components of successful learning. What makes these findings significant is the context. First, previous research in this area has focused on skills that are certainly important (e.g., writing and reading comprehension) but perhaps not with the same stakes attached to them as stitching a surgical patient. The skills taught in this study have the potential to be life-saving, and students report that this responsibility is forefront of their minds at all times. This suggests that even in high-stakes learning environments like medical school, the nature of training and feedback are important to skill development. Second, in the medical literature, skills training is emphasized while measures of student motivation are often missing.

## Limitations of the study

We note that there are limitations to the current study. First, as noted earlier, our sample size was relatively small. While consistent with previous research findings, caution should be taken in generalizing our results with 78 participants to other learning contexts in medical training and other areas of education. Studies similar to ours, however, consistently suggest that the interaction of teachers' feedback style and modeling approach significantly affects student learning. The present study, then, offers support for this ongoing line of research, third year clerkship students performing a novel knot tying task seemed to have greater success with a coping model and with process feedback. A second limitation to this study relates to ecological validity. Activities were conducted in a simulation center, participation was completely voluntary, and artificial materials were used (e.g., a knot tying box trainer). It is possible that the learning environment affected how participants engaged with the practice materials. Practical concerns drive the use of simulation centers in medical education. Training related expense, for example, is far less with simulation materials, which can be reused, than with cadavers. It is far safer, furthermore, for novice medical students to practice with artificial materials than with human subjects in clinical settings. Finally, we found it necessary to use multiple teachers for the training and data collection phases, which can cause inconsistencies in administrating the experiment and in collecting data. We sought to address this potential problem by observing each interaction between teachers and students and by providing teachers with ongoing training throughout the experiment. In subsequent research, our goal is to have one teacher for the entire data collection phase to ensure fidelity to treatment.

## Ethics statement

This study was carried out in accordance with the recommendations of “Boston University School of Medicine Institutional Review Board” with written informed consent from all subjects. All subjects gave written informed consent in accordance with the Declaration of Helsinki. The protocol was approved by the BUSM IRB with exempt status.

## Author contributions

MD was responsible for the interpreting the data and the write-up and collaborated with DK on the data collection and analysis. DK was primarily responsible for the design and data collection, and collaborated with MD on the analysis, and edited versions of the manuscript.

### Conflict of interest statement

The authors declare that the research was conducted in the absence of any commercial or financial relationships that could be construed as a potential conflict of interest.

## Appendix

### Laparoscopic knot tying survey

Medical Students differ in how confident they are about achieving on various assignments and activities during the course of their clerkships. In relation to the intracorporeal knot tying activity you just completed, rate how confident you are that you can achieve each of the following by indicating a probability of success from 0 (no chance) to 100 (complete certainty). The scale below is for reference only; you don't need to use only the given values. You may assign ANY number between 0 and 100 as your probability.

**Table:**

**0**	**10**	**20**	**30**	**40**	**50**	**60**	**70**	**80**	**90**	**100**
No Chance	Very Little Chance		Little Chance		50/50 Chance	Good Chance		Very Good Chance		Complete Certainty
1. _____Grasp the needle with the needle driver at the midpoint of the needle arc.										
2. _____Pass the needle through the two black dots on the penrose drain.										
3. _____Reverse the orientation of the tail and the needle.										
4. _____Loop the needle end of the suture twice around the needle driver.										
5. _____Cut the two suture limbs with laparoscopic scissors.										
6. _____Insert the needle driver into the dominant hand port.										
7. _____Secure the third and final knot.										
8. _____Insert the Maryland grasper into the dominant hand.										
9. _____Grasp the structure with a laparoscopic needle driver.										
10._____Grasp the needle with the Maryland grasper.										

Medical students also differ with respect to how satisfied they are with their performance on different learning activities. In relation to the intracorporeal knot tying activity you just completed, rate how satisfied you are with your performance. Again, the scale below is for reference only; you may assign ANY number between 0 and 100 ___________________.

**Table:**

**0**	**10**	**20**	**30**	**40**	**50**	**60**	**70**	**80**	**90**	**100**
	Not Satisfied		Somewhat satisfied			Pretty Satisfied				Very Satisfied

Finally, medical students often differ with respect to how they compare different learning activities with one another. Please rank order the following activities you may have already experienced from most interesting (1) to least interesting (6).

**Table:**

_____An anatomy lab exercise	_____A trauma/code simulation
_____A suture knot tying activity	_____A Student lead case presentation
_____A standardized patient-based simulation activity	_____An interesting lecture
